# Interventions to promote healthy nutrition and lifestyle and to tackle overweight and obesity amongst children in need in Europe: a rapid literature review

**DOI:** 10.3389/fnut.2024.1517736

**Published:** 2025-01-07

**Authors:** Anela Halilagic, Renos Roussos, Matzourana Argyropoulou, Vaios Svolos, Christina Mavrogianni, Odysseas Androutsos, Theodora Mouratidou, Yannis Manios, George Moschonis

**Affiliations:** ^1^Department of Food, Nutrition and Dietetics, School Allied Health, Human Services & Sport, La Trobe University, Melbourne, VIC, Australia; ^2^Department of Nutrition and Dietetic Sciences, School of Health Sciences, Hellenic Mediterranean University, Sitia, Crete, Greece; ^3^Department of Nutrition and Dietetics, School of Health Sciences and Education, Harokopio University of Athens, Athens, Greece; ^4^School of Medicine, Dentistry and Nursing, College of Medical, Veterinary and Life Sciences, University of Glasgow, Glasgow, United Kingdom; ^5^Lab of Clinical Nutrition and Dietetics, Department of Nutrition and Dietetics, School of Physical Education, Sports Science and Dietetics, University of Thessaly, Trikala, Greece; ^6^Institute of Agri-food and Life Sciences, University Research & Innovation Center, H.M.U.R.I.C., Hellenic Mediterranean University, Crete, Greece; ^7^La Trobe Institute for Sustainable Agriculture & Food (LISAF), La Trobe University, Melbourne, VIC, Australia

**Keywords:** obesity, overweight, children, adolescent, best practice, intervention

## Abstract

**Introduction:**

The global prevalence of overweight and obesity has reached epidemic proportions amongst children and adolescents, with approximately one third of children in Europe being overweight or obese. There is a higher likelihood for overweight and obesity reported for vulnerable groups of children, also known as children in need. As there is currently no knowledge of the best practice interventions for improving nutrition and physical activity habits amongst these vulnerable groups, the aim of this rapid review was to examine evidence that focused on best practice interventions to tackle overweight and obesity in children in need in Greece and/or other European countries.

**Methods:**

Studies were sought that presented methods and results of interventions and their evaluation. There were 989 results originally identified from database searches, which then reduced to 8 studies for inclusion following abstract and full-text screening processes. A narrative synthesis was conducted to present the characteristics and results of each study.

**Results:**

The only group of children in need that was identified was of low socioeconomic status (SES). There were several interventions identified that were successful across various countries in Europe, however there is a lack of best practice interventions to contribute to longer lasting solutions to preventing overweight and obesity.

**Conclusion:**

Our findings encourage the implementation of interventions that will be inclusive of children with vulnerabilities other than low SES, as well as the establishment of specific criteria guiding the design of best practice interventions.

## 1 Introduction

The global prevalence of overweight and obesity has reached epidemic proportions amongst children and adolescents (1). The rate of adolescent obesity has increased fourfold since 1990 and there was a recorded 390 million children and adolescents living with overweight and obesity in 2022 according to the World Health Organization (WHO) (2). When focusing on Europe specifically, the prevalence of overweight and obesity is 28% amongst girls and 31% amongst boys (3). Although specific countries in Southern Europe, including Greece, Italy, Portugal and Spain, have reported a decrease in children living with obesity, prevalence of overweight and obesity still remains high in these countries (3).

It has been reported that approximately one third of children living with overweight or obesity continue to follow a trajectory of high body mass index (BMI) into adulthood (4). Although the development of obesity is complex (5), excess body weight is a result of an imbalance in energy intake and expenditure, mainly through decreased physical activity (PA) and increased dietary energy intake (6). Childhood obesity is related to health consequences both short term and long term, and an overall increased risk of morbidity and mortality in adulthood (1).

Specific groups of children and adolescents, such as those living in low socioeconomic areas and those living with a disability, are more likely to be living with overweight or obesity than other children (7). Research has shown that adolescents living in socio-economically disadvantages areas often lack to meet recommendations and guidelines relating to healthy lifestyle behaviours (8). Children and adolescents from low socioeconomic status (SES) families are at higher risk for overweight and obesity compared to other children from higher income backgrounds (1, 9). Similarly, children with mobility limitations and intellectual or learning disabilities are also at greater risk for obesity development (10). Other groups of children that are at risk of poverty or social exclusion, and therefore at greater risk of developing obesity, include children residing in alternative care (i.e., orphanages or foster care), children with a migrant or minority ethnic background, including refugee children, and children living in precarious or uncertain family situations (11). Half of refugees globally are children, with migrants from low and middle-income countries at higher risk of obesity when settled in developed countries (12). Children that fall into these categories are known as children ‘in need’ and are the focus of this review.

The economic recession in Greece since 2008 has had a negative impact on the population and the standard of living, with poverty estimated to have increased by 100–200% (13). The health, and more specifically of the immigrant population in Greece, was one of the key concerns that arose from this time. SES factors have been shown to negatively affect health whilst unemployment adversely impacts both physical and mental health (13). Further to this, food insecurity negatively impacts children’s health and growth, whilst also being directly associated with obesity and poor nutrition quality (14). As a result of these unfavourable socioeconomic circumstances, there are short-term and long-term effects on children’s health (14).

SES plays a key role in shaping health behaviours, including dietary habits and PA levels (14). The behaviours associated with these factors, which are related to the positive energy balance leading to overweight development, are known as energy balance-related behaviours (EBRBs) (15). Socioeconomic inequalities have been identified across various countries and amongst all age groups, with unhealthier EBRBs being adopted by people with lower SES (16).

Behaviours which result in a positive energy balance are established early in life, with the preschool age being a key period for implementing interventions for behaviour change (4). Regarding adolescents, there are concerns about unhealthy dietary habits and low levels of PA (1). Adolescents of a lower SES have been found to more likely report lower consumption of fruits and vegetables, higher consumption of dietary fat and food with added sugars, whilst also being more likely to skip breakfast and perform less PA (16). In contrast, children and adolescents of higher SES have been shown to have higher fruit and lower soft drink consumption (1). The levels of maternal education and family income have been reported as the main socio-economic factors that have been inversely associated with PA levels amongst adolescents (17).

Reducing the SES-related inequalities in health behaviours has been identified as a priority for public health policy in Europe (1), given the effect on health and quality of life during childhood, and the heightened risk of obesity in adulthood (9). The European Commission has placed emphasis on varying diet quality, level of PA, and prevalence of obesity in relation to socioeconomic factors within and across countries (9). Further to this, interventions that are more likely to have higher feasibility, acceptance rates, and lower risk of issues during implementation, should fit within the unique setting and allow for local adaptation (6). This is further justified by differences in context such as healthcare, where the structure and organisation of healthcare varies widely across countries in Europe (18). Health promotion within schools is advocated by the WHO (19), where they can provide a supportive environment for implementing interventions and involving family and teachers (14). School is also viewed as a preferred setting because of the higher potential to reach children of all SES (16). The implementation of an intervention that promotes a healthy diet and PA is more comprehensive when it focuses on educating school children, provides a stimulating environment, and also by engaging parents and the community (6). A meta-analysis that focused on the effectiveness of educational interventions in preventing paediatric obesity found that programmes that involved both school and family settings and that lasted ≤1 year were the most effective for primary school aged children (20). WHO also recommends engaging young people in the process of designing and delivering interventions that focus on healthy lifestyles (8).

Although several interventions have been implemented and evaluated in Europe to tackle childhood obesity in the general population, there is currently no knowledge of the best practice interventions for improving nutrition and PA habits in vulnerable groups of children. Best practice or good Practice can be defined as a methodology that, through research and experience, has proven to reliably lead to a desired result (21).

The aim of this rapid review was to examine the evidence currently available in the literature that focuses on best practice interventions related to promoting healthy nutrition or lifestyle to tackle overweight and obesity in children in need, implemented in Greece and/or other European countries.

## 2 Methods

### 2.1 Eligibility criteria

This review sought studies that described interventions implemented amongst children in need in Europe, and that also presented outcomes and results following intervention. The population group that this review was focused on was children in need or children living under certain conditions that makes them more vulnerable. This includes children that come from low SES families, children who are homeless or experiencing housing deprivation, children living with a disability, children with mental health issues, children in alternative care (i.e., orphans or children living outside of family care), children with a migrant background or minority ethnic origin, refugee children, and children that are living in precarious family situations. The limitation placed on age was 0–17 years, which allowed for the inclusion of all stages of childhood and adolescence. There was no year of publication limit placed due to the possible limited amount of available literature on these children in need and health-promoting interventions. In addition to this, studies were not limited to best practice interventions to prevent further restrictions on literature. Regarding length of follow up, no minimum duration was set to prevent any further restriction on the scarce number of studies that have focused on the specific population group.

The primary outcomes of interest included measures of childhood overweight or obesity, such as anthropometric indices related to BMI, weight-for-age, BMI-for-age, and waist circumference, as well as EBRBs. Other potential outcomes included weight and body composition values. Studies were eligible to be included if they were written in English, involved human participants, and were peer reviewed. All study designs, except for literature reviews, were included however, reference lists were searched if literature reviews were identified.

Exclusion criteria included studies that were not conducted in Europe, studies that did not include children with a vulnerability as listed above, or studies with missing outcomes or results, such as if an intervention was described in a study but there has been no evaluation of the study to date. Identified studies that were only abstracts for conferences were also excluded.

### 2.2 Searches and sources

The databases that were searched to identify relevant studies were Medline, CINAHL, Web of Science, and Cochrane. Published and unpublished studies were both sought for with reference lists also reviewed to ensure that all possible eligible studies, that may not have been identified in the searches, were included in the review. The searches were completed on the 5th of May 2024. The search strategy was based on the PICO framework, which includes key words related to the population, the intervention, the comparison group, and the examined outcome(s). The studies that were sought were not required to include a comparison or control group, therefore this component was not included in the search strategy to avoid limiting the results. The search strategy has been presented in Table 1. The Boolean operators ‘OR’ and ‘AND’ were used to combine terms within each category and to combine each category, respectively.

**Table 1:** Search strategy used in Medline.

PICO	Keywords	
Population	1.	Child or Child* or Adolescen* or Pediatric
	2.	Greek or Greece or Europe or EU or South Europe
	3.	In need or Vulnerable or Homeless or Disab* or Mental health or Migrant or Refugee or Roma or Institutional care or Juvenile prison or Low socioeconomic or Low SES or Low education or Disadvantaged or Depriv*
	4.	1 and 2 and 3
Intervention	5.	Intervention or Initiativ* or Implement or Plan or Treatment or Approach or Best practice
	6.	School based or Child-care or Preschool or Family-based or Home or Health or Primary care or Community or Education or Multi-setting or Behavioural or Nutritional or Physical activity or Long-term or Outcomes
	7.	5 and 6
Outcome	8.	Obesity prevent* or Overweight prevent* or Healthy diet or Physical activity or Healthy food or Healthy eating or Active living or Healthy lifestyle
	9.	4 and 7 and 8

* refers to truncation and allows the database to search for all forms of a word.

### 2.3 Study selection

The initial database searches were conducted by one reviewer, AH. Once searches were completed, all results were exported to Endnote 21.3 (22) and Covidence.^1^ Endnote was used to store the references whilst Covidence was used to first remove all duplicates. The title and screening process then began where one reviewer, AH, excluded studies based on the eligibility criteria. The full-text screening process was conducted by two reviewers, AH and RR. Independent full-text screening was completed by both reviewers to exclude any further ineligible studies and to determine the final number of studies to be included in this review. Conflicts in decisions between reviewers was resolved by involving a third reviewer, GM, to make a final decision. Only one study author was contacted to request further study results, however this was not included due to unpublished results (8).

### 2.4 Data extraction

The data extraction process was completed by one reviewer, AH, with all extracted data inputted into an Excel spreadsheet set with a template of all required variables. The following variables were included: title, authors, date of study, study design, country/location, population vulnerability (i.e., low SES), sample size, sample age, sample sex, intervention, measures, and results. The selection of these variables was determined by the PICO framework, search strategy, and literature. The extracted data was then summarised into a separate table (Table 2) with only the details that were identified as most relevant to this review. The column titles in this table included study (authors, year, and study design), location, age and sex, sample size (n), vulnerability, intervention, measures, and results.

**Table 2:** Summary of studies.

	Sample						
Study	Location	Age and sex	*n*	Vulnerability	Intervention	Measures	Results
Borys et al. (2016). Prospective, 2-year	France, Bulgaria, Netherlands, Portugal, Greece, Romania, Belgium Community-based	6–9 years “Boys and girls represented almost 50% each sample”	921	Low SES SES level determined by educational level, employment status, and income position of parents.	The EPODE for the Promotion of Health Equity. Interventions focused on: 1. Promotion of water consumption 2. Promotion of an active lifestyle 3. Promotion of fruit and vegetables consumption 4. Promotion of an adequate sleeping behaviour. Community-based interventions tailored to the needs of the low socio-economic group.	EBRBs: - Fruit and vegetables consumption - Soft drinks/fruit juices and water consumption - Screen time - Sleep duration And their determinants Measured via parental questionnaire	Significant findings: ↑ Fruit frequency (Dutch low SES) ↓ Fruit juice amount (Romanian low SES) ↑ Screen time (Bulgarian high SES) ↓ Home availability of soft drinks (Portugal low SES) ↑ parental practices related to fruit juices consumption (Belgium, Greece and Portugal) Identified as best practice
De Bourdeaudhuij et al. (2011). Secondary data analysis of 3 intervention studies: Study 1: Haerens et al. (2007), clustered randomised controlled trial, 2-year Study 2: Simon et al. (2006, 2008), randomised, 4-year Study 3: Haerens et al. (2007), clustered randomised controlled trial, 3-month	Study 1: Belgium School-based Study 2: France School-based Study 3: Belgium School-based	Study 1: 13.1 +/−0.8 years Study 2: 11.7 +/− 0.6 years Study 3: 13.2 +/− 0.7 years	Study 1: 2840 Study 2: 954 Study 3: 139 (intervention), 142 (control)	Low SES, SES level determined by: Study 1: parents’ occupation classification Study 2: highest occupational category of either parent Study 3: educational level of adolescent	Study 1: ↑ opportunity for PA, PA content and sports materials provided to schools, Personalised, computerised PA advice for students, Parents involved in interactive meeting and provided with adult computer-tailored interventions. Study 2: ICAPS ↑ access to activities during breaks and after-school hours, Encouraging social support and providing environmental conditions that enable PA, Adapting times and places to increase accessibility, open participating, emphasis on fun, meeting with others, absence of competitive aspects. Study 3: After completing online questionnaires in the computer classes at school, personalised PA advice provided to students.	Study 1: PA measured via questionnaire and accelerometers Study 2: PA measured via questionnaire Study 3: PA measured via questionnaire	Stratified analyses did show some significant differences. Study 1: High SES: ↑ of 6 min/day self-reported PA (intervention w/ parental support) ↑ of 8 min/day school-related PA (intervention w/ parental support) Low SES: ↑ of 6 min/day school-related PA (intervention w/ parental support) compared to ↑ 4 min/day (intervention alone) Study 2: Both SES groups ↑ Supervised PA (intervention) ↑ Active commuting to and from school (both intervention & control) Study 3: High SES: ↑ of 4.5 min/day school-related PA (intervention) No significant intervention effects on total PA, leisure time sports, and leisure time active transport. Low SES: ↑ of 3 min/day active transport (intervention) Not identified as best practice
Dubuy et al. (2014). Prospective, 4-month	Belgium School-based	10–14 years Limited number of girls in intervention group led to their exclusion in further analyses	146 (intervention) 268 (control) (After removing girls)	Low SES Schools selected by football clubs based on number of ‘socially vulnerable pupils’.	‘Health Scores!’ Use of professional football players as a credible source for promoting health behaviours. Clinics at football clubs included eating healthy breakfasts, warm up session with players, and signing a lifestyle contract. School programme included providing free fruit, fruit & veg quiz, lesson on importance of drinking enough water, active playgrounds and activity breaks. Video messages and letters from players were provided to teachers to include in lessons.	Intake of fruits, vegetables, water, soft drink, and sweet and savoury snacks assessed via FFQ. PA levels (active transport and sport participating) assessed via questionnaire.	↑ Self-efficacy for having daily breakfast (*p* < 0.01) ↑ Positive attitude towards veg consumption (*p* < 0.10) ↓ Soft drink consumption (*p* < 0.001) No significant intervention effect found for sport participation. Not identified as best practice
Kastorini et al. (2016). Prospective, cohort study, 1 year	Greece School-based	3–12 years (children) & 13–18 years (adolescents) 48.1% boys 51.9% girls	3,941	Low SES SES determined via the Family Affluence Scale (FAS)	DIATROFI programme Free meals for students being high in fruit, veg, and protein, with exclusive use of olive oil. Education material encouraging healthy eating practices and PA was distributed to students and families throughout the year. Health-promotion events by nutrition experts and demonstrated by chefs were organised.	Lifestyle and dietary habits determined via questionnaire. Diet quality based on KIDMED score and food frequency data.	↑ KIDMED score in adolescent girls (*p* = 0.042) ↑ Consumption of milk, fruits, veg, and whole grain products increased for children and adolescents, boy and girls (*p* < 0.002) Factors indicating lower SES, such as foreign country of birth, lower education level, no income source, and high levels of food insecurity were associated with lower diet quality. Identified as good practice
Lien et al. (2014) Secondary data analysis of 3 intervention studies: Study 1: Haerens et al. (2007), clustered randomised controlled trial, 2-year Study 2: te Velde et al. (2008), clustered randomised controlled trial, 1 and 2-year Study 3: Murphy et al. (2011), clustered randomised controlled trial, 1-year	Study 1: Belgium School -based Study 2: Netherlands, Spain and Norway School-based Study 3: Wales School-based	Study 1: 11–15 years Study 2: 10–12 years Study 3: 9–11 years	Study 1: 2840 Study 2: 1245 Study 3: 4350	Low SES SES level determined by: Study 1: Parental occupation Study 2: Parental length of education Study 3: School socioeconomic position	Study 1: Teenage project Personalised computer-tailored advice regarding fat and fruit intake. Schools/teachers encouraged to arrange other activities (healthy breakfast). In group with parental support - parents received tailored advice for fat intake. Parents invited to interactive meeting. Changes in school to increase availability of healthy foods. Study 2: the Pro Children Study Classroom activities, personalised computer-tailored advice, a parent component (involvement through children’s homework, parental newsletters, and personalised tailored advice for adults) and provision of FV. Study 3: Breakfast provided before school every day, free of charge and healthy.	Study 1: Fat intake measured via FFQ Study 2: Fruit and vegetable intake measured via 24 h recall Study 3: Breakfast measured via dietary recall questionnaire	Study 1: Effect of intervention found amongst girls of low SEP only. ↓ Fat intake more amongst girls of intervention w/parental support compared to intervention alone (*p* = 0.046) or control (*p* < 0.001). Study 2: ↑ FV intake in both SES groups ↑ Vegetable intake in low SES group ↑ Fruit intake in high SES group Study 3: ↑ Consumption of healthy breakfast items was only found in low SES group Not identified as best practice
Manios et al. (2021). Cluster-randomised design, 1-year	Belgium, Bulgaria, Germany, Greece, Poland and Spain Kindergarten-based	3.5–5.5 years Intervention: 51.9% boys 48.1% girls Control: 52.0% boys 48.0% girls	3,997 (intervention), 2,271 (control)	Low SES SES determined via parental education	ToyBox Study 1-year intervention focused on: - Water consumption - Healthy snacking - Sedentary behaviour - Physical activity Kindergarten teachers were trained and delivered the programme in kindergartens	Diet assessed via FFQ - beverage consumption, snacking. Sedentary behaviour assessed via Primary Caregiver Questionnaire. PA assessed via step count (accelerometer). BMI percentile	Fruit juice consumption and computer/video game use decreased however no intervention effect on change in children’s BMI percentile. Identified as best practice
Tarro et al. (2018). Parallel-cluster randomised controlled, 10-month	Spain School-based	Children: 9–11 years Adolescents: 12–14 years (creators)	Intervention: 375 (children) 94 (adolescents) Control: 327 (children), 98 (adolescents)	Low SES SES determined via location of schools in low SES neighbourhoods	EYTO-Kids project Adolescent creators designed and implemented four social marketing activities for their younger peers.	Diet (FV consumption, soft drinks, sweets, and fast food consumption) PA and screen time Assessed via questionnaires	Fruit consumption and PA were maintained in children who consumed at least 1 fruit/day and spent at least 6 h/week doing PA. ↑ to 15.6 min/week of PA in girls in intervention compared to controls ↓ of % of girls who consumed sweets, soft drinks and fast food decreased significantly by 8.4, 14.5, and 5.9%. ↓ % of boys who had at least 2 h/weekday of screentime by 8.2%. Not identified as best practice
Verjans-Janssen et al. (2020). Quasi-experimental, 1 and 2-year follow up	Netherlands School-based	7–10 years 46% boys 54% girls	523	Low SES SES determined via location of schools in low SES neighbourhoods	KEIGAAF intervention Each school formed a working group that developed and implemented the intervention according to the needs of the children and possibilities within the community. Working groups were to implement a comprehensive approach of PA and healthy nutrition-promoting activities. Examples: new PA equipment during school recess, provision of water bottles to children, implementation of monthly after-school sports activities.	BMI z-score and EBRBs (sedentary behaviour, PA and nutrition) via anthropometry, accelerometers and questionnaires	↓ BMI z-score after 2 years compared to control group The intervention prevented an age-related decline in moderate-to-vigorous PA. Negative intervention effects were seen on sugar-sweetened beverages and water consumption at school, due to larger favourable changes in control group. Identified as best practice
Van Stappen et al. (2021). Cluster randomised controlled trial, 2-year	Belgium, Finland, Greece, Spain, Hungary, and Bulgaria School, community and family-based	8.1 +/− 1 years 51% girls 49% boys	1,329 (intervention) 1,089 (control)	Low SES SES determined via country’s income level and socioeconomic indices of each municipality	Feel4Diabetes intervention Goals were to ↑ water, FV, and breakfast consumption, ↑ PA, and ↓ sedentary time Family: Parents received counselling sessions and motivational guidance via text messages. School: Teachers received an information session and parents received newsletters Community: Families received communication regarding available infrastructure and health-related activities	Moderate-to-vigorous PA Screen-time behaviour Consumption of water, soft drinks, and juices containing sugar. Fruit and vegetables, unhealthy snacks and breakfast All measured via questionnaire completed by parents	No significant effect on water. Salty snacks, fast food, and breakfast consumption, and screen time amongst children (*p* > 0.05) ↑ FV consumption amongst Belgian children after 2 years (*p* = 0.01) ↓ Consumption of sweets across all participating countries after 2 years (*p* = 0.01) ↓ Soft drink and juice consumption amongst Spanish children after 1 year (*p* = 0.04) ↑ Moderate-to-vigorous PA across all countries (*p* = 0.03)

### 2.5 Data synthesis

A summary of the selected studies in this review was prepared in the form of a narrative synthesis. This synthesis included highlighting the main similarities and differentiating factors between studies, mainly focusing on interventions and results. Due to the nature of a rapid review and the heterogeneity of the included studies, a meta-analysis was not feasible nor appropriate.

## 3 Results

### 3.1 Study selection

**Figure 1:**
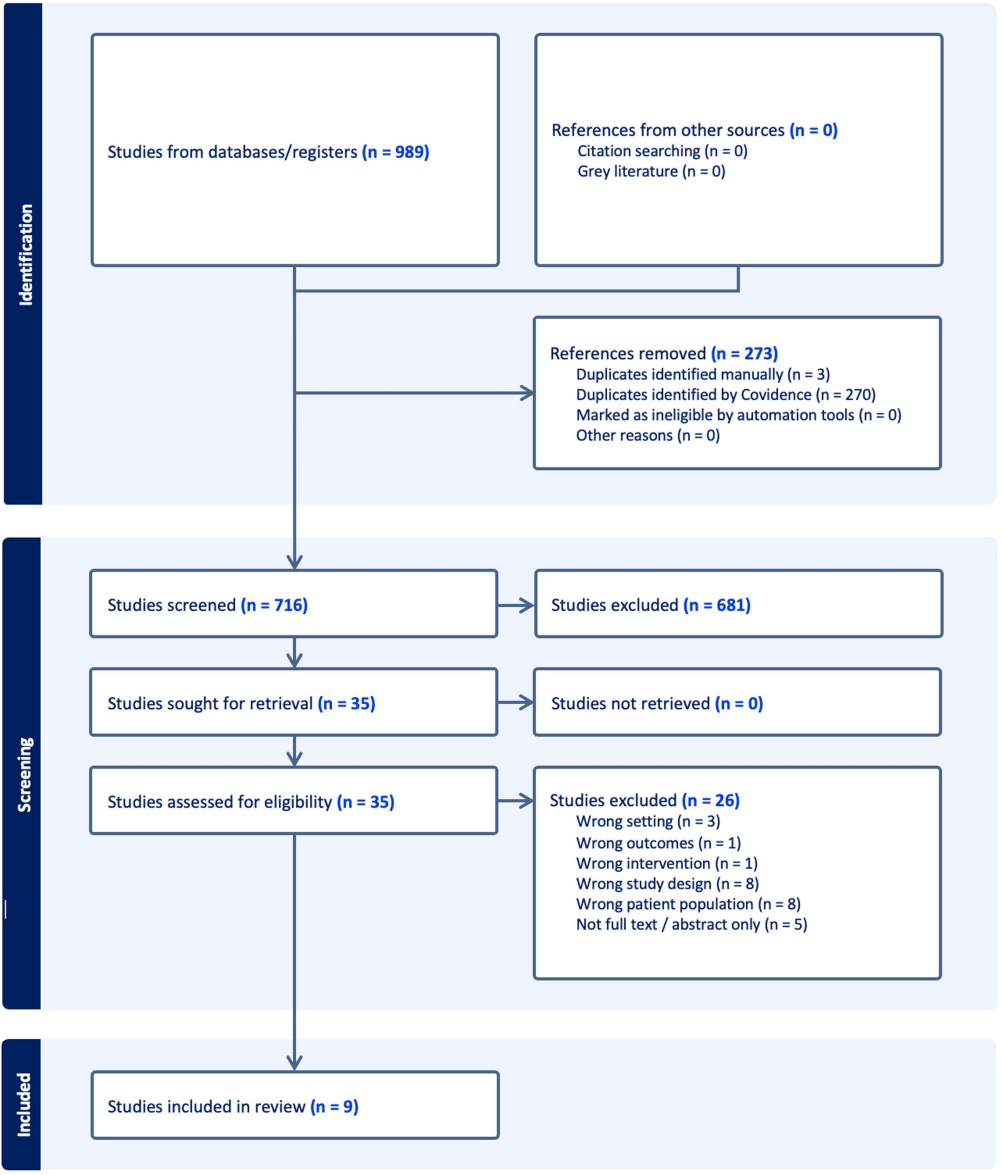
Search strategy used in Medline.

Upon completion of the initial database searches there were 989 studies identified. The removal of 273 duplicates in Covidence (270 automatically and 3 manually) left 716 studies left to undergo title and abstract screening. Following this screening process, a further 681 studies were excluded leaving 35 studies to have their full texts reviewed. Studies were included or excluded based on the eligibility criteria, resulting in 8 studies included and 26 studies excluded due to wrong patient population (i.e., not relevant), study design, setting, intervention, or unavailability of full text (Figure 1). The final number of studies was 9, including 3 prospective cohort, 3 clustered-randomised controlled, 1 quasi experimental, and 2 secondary data analyses. Within the secondary data analyses, three clustered-randomised controlled studies were included in each. For the purpose of describing the characteristics and results of the study interventions, the six studies identified in the secondary analyses will be referred to individually, therefore resulting in 13 total studies.

### 3.2 Study characteristics

The included studies were conducted across different countries in the European region ranging from Wales to Romania. Nine studies were completed in single countries, with four in Belgium (1, 23–25), one in France (26, 27), one in Spain (28), one in the Netherlands (6), one in Wales (29), and one in Greece (14). The remaining four studies were multi-centred; the EPODE study was conducted in France, Bulgaria, Netherlands, Portugal, Greece, Romania and Belgium (9), the Pro Children study was conducted in the Netherlands, Spain and Norway (30), the ToyBox study was conducted in Belgium, Bulgaria, Germany, Greece, Poland and Spain (4), and the Feel4Diabetes study was conducted in Belgium, Finland, Greece, Spain, Hungary, and Bulgaria (31).

The ages of children and adolescents in the included studies ranged from 3 to 18 years. The youngest sample group was aged 3.5–5.5 years (4), with another study also including this same age group but further extending through to adolescence, 3–18 years (14). Several studies included children from middle childhood, with 6–9 years (9), 8.1 +/− 1 years (31), 7–10 years (6), 9–11 years (28, 29), and 10–12 years (30), with the remaining studies including children of early adolescent age; 9–14 years (28), 10–14 years (1), 11–15 years (24), 11.7 +/− 0.6 years (26), 13.1 +/− 0.8 years (23), and 13.2 +/− 0.7 years (25).

The sample sizes, or number of children that participated in the studies, ranged from 281 (25) to 6,268 (4). One study reported that there were a limited number of girls in the intervention group, which led to their exclusion in further analyses, resulting in their final sample size (*n* = 414) consisting of only boys (1). The remainder of studies included both boys and girls, however not all reported the percentages or numbers of each. Of those that did (4, 6, 9, 14, 31), they were on average equal between boys and girls.

### 3.3 Vulnerabilities and settings

When focusing on the vulnerability of each population sample, every study included children of low SES. The methods of determining how children were categorised into the low SES category varied between studies. Three studies determined SES status based on parental occupation (23, 24, 26), two based on parental level of education (4, 30), one on family affluence scale (14), and one on parental education level, employment status, and income (9). One study determined SES level via the adolescents’ education level (25). Adolescents that were enrolled in general education (focusing on theory) were categorised as high SES, whilst those enrolled in vocational education (focusing on practical skills) were categorised as low SES (17, 25). One study identified schools as low SES based on the number of socially vulnerable students (1), and the remaining four studies identified schools as low SES based on school socioeconomic position (29) or their location in low SES neighbourhoods (6, 28, 31). Eleven studies were school-based, whilst one was based in kindergartens (4), and one was community-based (9).

### 3.4 Interventions

The interventions described in each study were diverse and distinct in their focus, delivery, and involvement of different groups of contributors. The EPHE (EPODE for the Promotion of Health Equity) project’s objective was to analyse the value of community-based approaches based on EPODE methodology to reduce inequities associated with childhood obesity and related determinants (32). This project focused on four EBRBs: promotion of water consumption, an active lifestyle, fruit and vegetables consumption, and adequate sleeping behaviour (32). Following the completion of the EPHE parental questionnaire, the EPHE communities developed community-based interventions tailored to the needs of their SES group (32). Changes in EBRBs were assessed at a 2-year follow-up following implementation of interventions in each country.

De Bourdeaudhuij et al. (17) completed secondary analyses of three school-based studies to determine whether PA interventions in European teenagers were equally effective in low and high SES groups. The first study to be included was 2-year RCT that aimed to increase PA opportunities by providing PA content/sports materials to schools, provided personalised, computerised PA advice for students and parents, whilst also involving parents in an interactive meeting (23). The second study to be included was ICAPS, a 4-year study that increased access to activities during breaks and after-school hours, encouraged social support and provided environmental conditions that enable PA (26). The third study included in these analyses was a 3-month RCT that provided students with personalised PA advice after completing online questionnaires in classes at school (25).

‘Health Scores!’ was a prospective, 4-month study that used professional football players to promote healthy behaviours (1). This included holding clinics at football clubs where students were provided with a healthy breakfast, took part in a warm-up session with players, and signed a ‘lifestyle contract’ (1). The school programme included providing free fruit, a fruit and vegetable quiz, a lesson on the importance of drinking enough water, and active playground and activity breaks. Video messages and letters from players were also provided to teachers to include in lessons (1). The DIATROFI programme supported students in primary and secondary schools and provided free, healthy meals for students in Greece, whilst also organising health-promotion events by nutrition experts and chefs. Education material encouraging healthy eating practices and PA was also distributed to students and families throughout the year (14).

Lien et al. (16) conducted the European TEENAGE project by conducting secondary data analyses to explore subgroup effects of low and high SES in three previous intervention studies. The first study that was analysed was a 2-year RCT that provided personalised computer-tailored advice regarding fat and fruit intake for students (24). Schools increased availability of healthy foods whilst also being encouraged to arrange other activities, such as healthy breakfasts (24). Parental support was also provided through invitations to take part in an interactive meeting and provided with tailored advice for fat intake (24). The second study that was included was the Pro Children study that involved classroom activities, personalised computer-tailored advice, a parent component (involvement through children’s homework, parental newsletters, and personalised tailored advice for adults) and provision of fruit and vegetables (30). The third study involved providing a healthy breakfast before school every day, free of charge (29).

The ToyBox study was conducted using an intervention, with a duration of one school year, that focused on water consumption, healthy snacking, sedentary behaviour, and physical activity (4). Kindergarten teachers were trained and delivered programmes in kindergartens to the children using the standardised framework for planning and implementing the intervention (4). The EYTO-Kids project involved both children and adolescents, where adolescents were allocated the role of creators in designing and implementing four social marketing activities for their younger peers (children group) (28). Activities were based on physical activity, popular games and TV shows, including trivia. Students that won were awarded with stickers, medals and sashes, and provided with fruit and vegetable tastings, and general recommendations for healthy lifestyles for all students (28).

The Feel4Diabetes study was based on an intervention focusing on families at risk of developing type 2 diabetes who were also considered to be living in a vulnerable or low SES area. The goals were to increase the consumption of water, fruits and vegetables, and breakfast, whilst also increasing physical activity and reducing sedentary time. Each country was advised to make adaptations to the local needs and circumstances. The intervention focused on families, school, and community. Parents received counselling sessions and motivational guidance via text messages. Teachers in schools received an information session whilst additionally providing parents with newsletters. Finally, families also received communication regarding available infrastructure and health-related activities in their area (31).

The final study was based on the KEIGAAF intervention where each involved school formed a working group that developed and implemented the intervention according to the needs of the children and possibilities within the community (6). Working groups were to implement a comprehensive approach of PA and healthy nutrition-promoting activities, some of which included new PA equipment during school recess, provision of water bottles to children, and implementation of monthly after-school sports activities (6).

### 3.5 Outcome measures

The measures of outcomes amongst all the studies included in this review come under several categories, however majority can be summarised into EBRBs, related to diet and PA. EBRBs and their determinants were measured by parental questionnaire (6, 9, 31). Another study specifically assessed beverage consumption and snacking behaviours via FFQ completed by parents (4). Lifestyle and dietary habits were also determined by questionnaire, with diet quality based on KIDMED score and food frequency data (14). PA was measured by questionnaires alone (1, 25, 26, 28), accelerometers alone (4), and by questionnaires and accelerometers combined (23). Sedentary behaviour was measured via a primary caregiver questionnaire (4), with screen time also measured via another questionnaire (28).

Dietary intake was measured using several methods and assessing several components of diet. More specifically, groups included intake of fruits, vegetables, water, soft drinks, and sweet and savoury snacks assessed by FFQ (1) and questionnaires (28), fat intake assessed via FFQ (24), and fruit and vegetable intake only measured via 24 h recall (30). Breakfast consumption alone was measured in one study via a dietary recall questionnaire (29). Anthropometry was used to calculate BMI z-score (6) and BMI percentiles (4) in the KEIGAAF and ToyBox studies.

### 3.6 Diet outcomes

Several interventions resulted in findings related to diet outcomes, of which those that were significant are presented below. The study by Borys et al. (9) was conducted across seven countries and found an increase in fruit consumption frequency in the Dutch low SES group, a decrease in amount of fruit juice consumption in the Romanian low SES group and decrease in home availability of soft drinks in the Portuguese low SES group. It also found an increase in parental practices related to fruit juice consumption in Belgium, Greece, and Portugal (32). The ‘Health Scores!’ study reported findings including increased self-efficacy for having daily breakfast (*p* < 0.01) and decreased soft drink consumption (*p* < 0.001) (1). Similarly, the ToyBox study identified a decrease in fruit juice consumption (4). Following the implementation of the DIATROFI programme, there was an increase in KIDMED score in adolescent girls (*p* = 0.042) and an increase in consumption of milk, fruits, vegetables, and whole grain products for children and adolescents, both boys and girls (*p* < 0.002) (14). It was also highlighted that factors indicating a lower SES were associated with lower diet quality (14). The Feel4Diabetes study identified an increase in fruit and vegetable consumption amongst the Belgian children after 2 years (*p* > 0.05), a decrease in consumption of sweets across all participating countries after 2 years (*p* = 0.01), and a decrease in soft drink and juice consumption amongst Spanish children after 1 year (*p* = 0.04) (31).

The secondary data analysis by Lien et al. (16) also identified several findings within the three analyses. The first highlighted that the effect of intervention was found only amongst girls of low SES and this included a greater decrease in fat intake amongst girls of intervention with parental support compared to intervention alone (*p* = 0.046) or control (*p* < 0.001) (16). The second study identified increases in fruit and vegetable intake amongst both low and high SES groups (16). The final study within this secondary analysis found an increase in the consumption healthy breakfast items only within the low SES group (16). EYTO-Kids project found that children who consumed at least 1 fruit per day maintained this throughout the study (28). This study also highlighted significant decreases in the percentage of girls who consumed sweets, soft drinks and fast food by 8.4, 14.5, and 5.9%, respectively (28). Within the KEIGAAF study, negative intervention effects were seen on sugar-sweetened beverages and water consumption at school, due to larger favourable changes in control group (6).

### 3.7 Physical activity, sedentary behaviour and BMI-related outcomes

The secondary data analysis by De Bourdeaudhuij et al. (17) identified several significant findings following stratified analyses. In its first study, an increase of 6 min/day of self-reported PA and 8 min/day of school-related PA was observed within the intervention group with parental support in the high SES group (17). In comparison, the low SES group saw an increase of 6 min/day of school-related PA within the intervention group with parental support, compared to 4 min/day with no parental support (17). The second study found an increase in both SES groups in supervised PA (intervention group) and active commuting to and from school (both intervention and control groups) (17). The third study in this secondary analysis found that within the high SES group there was an increase of 4.5 min/day of school-related PA (intervention group) and within the low SES group there was an increase of 3 min/day of active transport (intervention group) (17). There was an increase of 15.6 min per week of PA in girls in the EYTO-Kids intervention group compared to controls (28). Verjans-Janssen et al. (2020) reported that the KEIGAAF intervention prevented an age-related decline in moderate-to-vigorous PA. In contrast, the ‘Health Scores!’ study found no significant intervention effect for sport participation (1). Finally, the Feel4Diabetes study found an increase in moderate-to-vigorous physical activity across all countries involved (*p* = 0.03) (31).

There was an increase in screen time found amongst the Bulgarian high SES group following the EPHE intervention (32). Additionally, there was a lower increase in computer/video game use was found amongst children in the ToyBox study following intervention, compared to the controls (4). The EYTO-Kids study identified a decrease of 8.2% of boys who had at least 2 h/weekday of screentime (28).

Following the ToyBox intervention implementation, no intervention effect on change in children’s BMI percentile was identified (4). In contrast, following the KEIGAAF intervention there was a decrease in BMI z-score after 2 years compared to control group (6).

## 4 Discussion

The age range of children in the selected studies demonstrates a wide spread across childhood, ranging from toddlers to adolescents. This distribution has allowed for comparisons to be made between settings (i.e., childcares, schools), interventions, and outcomes based on age. The countries that the studies were implemented in were Belgium, Finland, France, Hungary, Spain, Netherlands, Wales (UK), Greece, Bulgaria, Portugal, Romania, Norway, Germany, and Poland, and all except one were considered as high-income countries at the time of each study, with Belgium considered as an upper-middle income country (33). This overwhelming representation of high-income countries, and lack of representation of low- and middle-income countries, is likely due to disparities in human resources, grant funding and higher education opportunities (34). The majority of studies included in this review, more specifically two-thirds, were based on a cluster-randomised controlled design, with the remaining including prospective cohort and quasi-experimental. These study designs serve as high levels of evidence and therefore provide overall strength to the quality of evidence in this rapid review.

### 4.1 Socioeconomic status

As stated by the WHO, health and illness follow a social gradient where the lower the socioeconomic position of a group of people, the worse the health regardless of the income level of the country (35). Socioeconomic position may also be referred to as socioeconomic level or status (SES), and the ways in which how this was determined within the studies included in this review varied drastically. For those studies that categorised SES based on the parental data, only one took into consideration multiple factors which included parental education level, employment status, and income (9). The remaining of those were based on a single factor, whether that was occupation or level of education, potentially reducing the accuracy of the SES categorisation. A study investigating different approaches to measuring SES highlighted that there is a risk of subjective rating, especially when occupations of individuals do not match directly to job titles presented by a specific SES measure (36). The differences in status of specific occupations between countries is also another factor highlighted as another important consideration when basing SES solely on occupation (36). Other methods used to determine SES included adolescents’ education level, the number of socially vulnerable students within schools, and the SES of schools or neighbourhoods, further highlighting the potential differences in actual SES and difficulty of comparing study findings.

This review intended to capture a range of different groups that would classify as children in need, some of which include refugees, children with disabilities, and those living within institutions (i.e., orphanages). Contrastingly, only one specific vulnerable population was identified and that was children of low SES. The representation of children of low SES proves as a great opportunity for comparison of findings, however the lack of other groups of vulnerable children is a potential concern given their high risk of poverty and social inclusion, and therefore risk of obesity (11).

### 4.2 Follow-up and involvement

Other than the expected differences in specific health-related interventions amongst studies, two key factors that we would like to highlight are the differences in duration of follow up and involvement of parents and teachers. The follow up length varied between studies from 3 months to 4 years. There are several advantages of longer follow up durations which include the ability to assess the effectiveness of the interventions across later stages in life, the ability to examine the processes of disease development (i.e., obesity), and the ability to measure more accurate benefits and costs of an intervention (37). The difference in follow up between studies proves a barrier to conducting in-depth comparisons of results.

The involvement of parents and teachers in interventions also varied between studies, ranging from newsletters and tailored physical activity advice for parents, to kindergarten teachers being trained to deliver intervention programmes. When focusing specifically on teachers, it has been described that they play a significant role in the promotion and implementation of school-based health interventions (38). School intervention programmes that involve parents appear to be more successful, however the type and extent of involvement can vary with some evidence stating that providing parental information alone is insufficient (39). Given that twelve of the thirteen interventions in this review were school or kindergarten-based, we found that both teachers and parents were involved in different ways in most, therefore it is unclear if their involvement was a significant contributing factor to the outcomes achieved. Other than parents and teachers, it is also vital to identify the importance of social workers, governmental, and non-governmental organisations amongst these studies that supported the implementation of interventions amongst the children in need.

### 4.3 Outcome measures

The outcomes of interest and those that have been presented in this review include EBRBs, mainly diet and PA, amongst others. The outcomes varied between studies and were measured using several different methods. Methods for measuring EBRBs included parental questionnaires and FFQs, with methods for measuring diet ranging from 24 h recalls to questionnaires, and finally, methods for PA included questionnaires and accelerometers.

The different instruments used to measure these outcomes demonstrate an inconsistency in methods and therefore potentially an inconsistency in the results and the ability for direct comparisons. An example of this is that a 24 h recall only collects dietary data over the previous 24 h, which could be greatly impacted by several factors such as day-to-day variation and therefore it is not representative of the usual diet. In comparison, a FFQ collects data about frequency and often portion sizes about food and beverage consumption over a longer period of time, typically a month or year (40).

### 4.4 Targeting adolescents

When focusing on the differences in interventions based on the ages of the children, some consistent factors were identified. Successfully engaging and communicating with the target population are essential for an effective intervention (1), and several studies have demonstrated this planning. For the pre-adolescent and adolescent-aged groups, the inclusion of professional athletes, computerised components, and social marketing activities were incorporated. As proven in marketing strategies, there is evidence of the effectiveness on the use of celebrities or sports heroes to deliver specific messages (1). Positive diet-related outcomes were identified in the specific study that involved football players, however a need for further research on the use of football players to promote a healthy diet and PA in girls was identified (1).

Regarding social marketing, peer-led techniques have been demonstrated to be effective strategies (28). WHO also recommends engaging young people in the process of designing and delivering interventions (41). One study that incorporated this had adolescents develop social marketing activities for their younger pre-adolescent peers, and found some improvements in physical activity levels (28). Although there were some behaviour-related improvements in the group that the activities were targeted towards, the adolescent peers maintained their original behaviours even after designing and implementing these activities. A possible explanation for this was provided by the authors and was that the adolescents were not the target population and they were only aiming to improve the lifestyles of the younger children (28).

### 4.5 Effective components

Specific components of interventions related to diet and physical activity have been identified across several studies that have contributed to positive health-related outcomes. Increases in PA levels were the result of several interventions, some of which included increasing opportunities for PA (during and after school), providing PA content and sports equipment, personalised computerised PA advice, placing emphasis on fun and social inclusion, and incorporating peer-led activities. For outcomes that related to improvements in dietary intake and quality, components that were identified as contributing to these interventions include providing free meals, breakfasts, or water bottles, providing education materials and computerised dietary advice, organising health-promotion events led by experts or chefs, implementing peer-led activities, and incorporating athletes.

### 4.6 Best practice

A ‘best practice’ intervention, also known as ‘good practice’, is defined as an intervention that has demonstrated evidence of effectiveness and that is also likely to be replicable in other settings (42). Best practice interventions have been implemented in real-life settings and therefore are a valuable source of evidence on effective interventions (42). Only four studies included in this review have previously been identified as best or good practice interventions. These include the ToyBox study, KEIGAAF intervention, and Feel4Diabetes intervention as best practice and the DIATROFI programme as good practice. An outcome of a best practice intervention is that it can be implemented in other settings, and the ToyBox study has demonstrated this throughout several other countries. Due to its high impact, the ToyBox intervention is currently expanding to Argentina, Belgium, Ecuador, Germany, Italy, Malaysia, Malta, New Zealand, Nicaragua, Poland, South Africa, Scotland, Spain and the UK. Although the aim of this rapid review was to focus on best practice interventions, the inclusion criteria was not limited to this term, but rather focused on all interventions also targeting children in need, due to the predicted low number of studies that would have been identified.

### 4.7 Strengths and limitations

This rapid review presents with several strengths and limitations outlined below. The purpose of a rapid review is for findings to be presented in a timely and resource-efficient way and to accelerate the systematic review process (43). Although rapid reviews provide less in-depth detail, any deviations from traditional methods of systematic reviews should be reported. Strengths of rapid reviews include timely reporting of summaries of literature and an increase in uptake of research findings in clinical practice or policy development (43). An additional strength of this review is the inclusion of several processes associated with traditional systematic reviews, such as searching several databases and the use of two reviewers to conduct full-text screening, improving the quality of this review. The overall quality of the studies included is another strength to be highlighted, as two-thirds were cluster-randomised controlled studies.

Publication bias is a potential limitation of this review due to there being no unpublished studies identified or selected amongst an already scarce number of studies. This may have included studies that were in progress but did not have a full set of results to present, and therefore excluded from this review. The rapid nature of this review and heterogeneity amongst studies did not permit a meta-analysis which, like systematic reviews, would have improved the quality of results. The countries that studies were conducted in were mainly high-income countries, meaning that there is a potential limitation to the applicability of these findings to low- or middle-income countries. The focus location was European countries, which recognisably has an extensive range of languages other than English, therefore another likely limitation would be that literature searches were only conducted in English. This may have limited the results if there were other studies in other languages that would have been deemed eligible.

Several differences, such as methods of determining SES, the ages of children, interventions, and duration of follow-up, were an additional barrier which led to difficulty conducting direct comparisons and reaching valid conclusions. Additional differences between countries, including healthcare and education, is another factor that was likely contributing to the effectiveness of interventions, and limits the ability to directly compare. The resources in each country’s health system vary, with Germany’s healthcare expenditure being the highest in Europe, and even amongst the highest globally (18), with France, Italy, and Spain following (44). A final limitation to mention is that it is unknown whether the children that participated in the studies of this review would also fall within other categories of vulnerabilities. If the focus of the current studies were to categorise children or schools into SES categories, it is not known whether other vulnerabilities, such as refugee status for example, were also recorded or taken into consideration.

### 4.8 Implications and recommendations

Our findings align with previous publications that have highlighted the importance of implementing interventions to improve nutrition and PA habits amongst vulnerable groups of children to reduce overweight and obesity. The findings from this review summarise the interventions and outcomes related to only children of low SES. Due to the limited number of studies that are focused on children with other vulnerabilities, such as children with disabilities or of migrant background, it is recommended that future research focuses on these other children in need. It is known that vulnerable children, as outlined in the introduction, are at higher risk of developing overweight or obesity, and therefore should be highlighted as a priority during development and implementation of health-promoting interventions.

This summary of the current research serves as a basis for future planning of intervention implementation as it highlights key components and significant findings that could be applied in future intervention programmes targeting children in need. The current gaps in research are further highlighted in this review; there is a minimal number of interventions that have been deemed best practice and from the interventions that been applied to vulnerable groups of children, this has solely focused on children of low SES, excluding a large group of children in need. In addition to this, the current studies have been based in high-income countries and it is unknown if the effective interventions would also be feasible and applicable to vulnerable children in low- or middle-income countries. Any future implementation of interventions should consider the country-specific healthcare and education systems to improve practicality and effectiveness. Further research and expansion of best practice interventions is encouraged to answer further questions and fill the gaps identified.

## 5 Conclusion

Although there has been some success related to several interventions across different countries, there is still a lack of best practice interventions which would contribute to a longer lasting solution to preventing overweight and obesity, especially in vulnerable population groups such as children in need. The findings of the current rapid review encourage the implementation of interventions that will be inclusive of children with vulnerabilities other than low SES, as well as the establishment of specific criteria guiding the design of best practice interventions.

## Footnotes

^1^ https://www.covidence.org
